# A synergistic CoO/MXene heterostructure anode with facilitated interfacial charge transfer for high-rate micro lithium-ion batteries

**DOI:** 10.1038/s41378-026-01246-9

**Published:** 2026-05-12

**Authors:** Bingmeng Hu, Hanjing Wei, Hui Zhou, Titao Fang, Hailong Wang, Shixin Wang, Jinyang Han, Chenpeng Huang, Xiaoming Zhang, Xiaohong Wang

**Affiliations:** 1 School of Science, Minzu University of China, Beijing, China; 2 School of Information Engineering & Key Laboratory of Ethnic Language Intelligent Analysis and Security Governance of MOE, Minzu University of China, Beijing, China; 3 Department of Electrical and Systems Engineering, University of Pennsylvania, Philadelphia, PA USA; 4 Optoelectronics Research Center, Minzu University of China, Beijing, China; 5 School of Integrated Circuit, Tsinghua University, Beijing, China

**Keywords:** Engineering, Nanoscience and technology

## Abstract

Micro lithium-ion batteries (MLIBs) are promising power devices for miniaturized intelligent terminals, but are limited by low energy density and poor cycling stability. Among potential anodes, cobalt oxide (CoO) offers a high theoretical capacity while suffering from low electrical conductivity and structural degradation. Herein, we propose a synergistic 0D-2D CoO/MXene heterostructure anode with uniformly anchored CoO nanoparticles on Ti_3_C_2_ MXene nanosheets, enabling facilitated interfacial charge transfer and high-rate performance for advanced MLIBs. In this architecture, the nanosized CoO particles alleviate pulverization, shorten ion diffusion pathways, and amplify active sites, while the conductive, robust MXene framework facilitates electron conduction, ion transport, and accommodates mechanical strains. The intimate CoO-MXene vdW interface further enables synergistic charge coupling, leading to accelerated interfacial kinetics and enhanced electrochemical stability. The heterostructure is fabricated by a facile solvothermal strategy involving electrostatic adsorption of Co^2+^, MXene-guided nucleation, and oriented growth, which effectively suppresses CoO aggregation and MXene oxidation. Interfacial characterizations verify noncovalent vdW coupling, confirming intimate atomic contact and excluding covalent bonding features. As a result, the CoO/MXene 0D-2D van der Waals heterostructure achieves an ultrahigh reversible capacity of 1282.3 mAh g^−^^1^ after 100 cycles. It also exhibits an outstanding rate capacity of 731.7 mA h g^−^^1^ at 0.8 A g^−^^1^ for over 300 cycles. Notably, when the current density is returned to 0.1 A g^−^^1^ after high-rate cycling, the capacity recovers to 1111.6 mAh g^−^^1^, demonstrating remarkable structural integrity and electrochemical reversibility. Furthermore, density functional theory (DFT) calculations analyzed that the MXene-CoO heterostructure enhances conductivity through increased density of states near the Fermi level, optimizes interfacial charge-transfer kinetics to strengthen Li adsorption, and lowers Li⁺ diffusion barriers, collectively contributing to the improved rate performance and cycling stability. This work establishes a novel and controllable strategy for constructing 0D-2D vdW heterostructures and provides mechanistic insights into structure-property relationships, offering a practical pathway toward high-performance anodes for MLIBs applications.

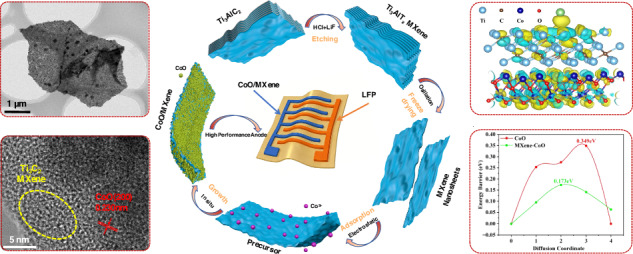

## Introduction

With the continuous advancement of artificial intelligence of things (AIoTs) and microelectromechanical systems (MEMS), miniaturized intelligent terminals such as implantable medical devices, self-powered sensors, smart dust, and micro drones have imposed increasing demands on their integrated energy sources^1–3^. As scaled-down versions that leverage the success of mainstream lithium-ion batteries, micro lithium-ion batteries (MLIBs) have emerged as highly promising energy storage devices for advanced microsystems^4,5^. However, as the device size decreases, the limited footprint area restricts the loading of active materials, resulting in low capacity. Moreover, the coupled issues of sluggish ion/electron dynamics and electrode structural instability are exacerbated under high-rate cycling, leading to rapid and significant capacity fade. Consequently, MLIBs still suffer from inadequate capacity and poor cycling stability at high rates, which represent major technical bottlenecks in the development of the energy electronics^6–8^.

Among the various approaches to enhancing the performance of MLIBs, optimizing anode materials has consistently been one of the most effective strategies. Transition metal oxides (TMOs) have attracted extensive attention as promising anode candidates due to their high theoretical specific capacity and relatively low cost^9–14^. As a representative TMO, cobalt oxide (CoO) stores lithium ions through a reversible conversion reaction, delivering a theoretical capacity as high as 716 mA h g^−^^1^, making it a highly promising anode material^15,16^. However, the practical application of CoO is hindered by structural degradation during cycling^17,18^ and poor electrical conductivity^19^, leading to rapid capacity fading and poor rate performance.

To overcome these intrinsic limitations of CoO, rational structural design has emerged as an essential strategy to improve charge transfer kinetics and structural stability. These design strategies can be categorized into two complementary approaches: intrinsic nanostructuring of CoO and the construction of CoO-based composite architectures. In the former approach, engineering CoO into nanoscale morphologies (such as nanoparticles, nanospheres, hollow nanocubes, and nanoflakes) can effectively shorten Li⁺ diffusion pathways, increase electrochemically active sites, and alleviate the mechanical stress associated with volume expansion during cycling, thereby mitigating pulverization and improving cycling stability^20,21^. Nevertheless, the nanosizing approach alone often induces particle agglomeration and unstable solid-electrolyte interface formation upon prolonged cycling, compromising the long-term durability of the anodes. In contrast, composite structure design integrates CoO with conductive scaffolds to overcome its intrinsic deficiencies in electronic conductivity and mechanical robustness. Conductive matrices such as carbon nanotubes, graphene, and reduced graphene oxide have been widely employed to construct CoO-based composites, forming interconnected networks that facilitate electron transport and buffer mechanical strain during lithiation/delithiation^22–24^.

Constructing heterostructures has proven effective in promoting charge transfer and stabilizing electrode structure. In particular, van der Waals (vdW) heterostructures formed between CoO and conductive substrates offer a promising configuration to facilitate interfacial ion/electron transport, minimize interfacial stress to maintain structural stability, and exploit synergistic effects between components. Among various 2D materials, MXenes, particularly Ti_3_C_2_, have demonstrated exceptional potential as building blocks owing to their metallic conductivity, rich surface chemistry, mechanical flexibility, ultrafast lithium-ion diffusion (10^−^^10^–10^−^^9^ cm^2^ s^−^^1^), and low diffusion barrier (0.07 eV), which are essential attributes for high-rate electrodes^25,26^. Recent studies have revealed that the synergistic interaction enhances electronic conductivity and reversible capacity^27,28^. Moreover, innovative structural and interfacial designs in vdW-integrated electrodes have been shown to effectively mitigate volume fluctuation and preserve mechanical integrity during lithiation/delithiation, resulting in improved long-term cycling stability^29,30^. However, existing synthesis strategies often fail to achieve uniform dispersion of CoO or full utilization of the MXene interlayer space, thereby limiting interfacial charge transfer efficiency and rate capability^31,32^. More importantly, the charge-transfer dynamics and interfacial coupling mechanisms within CoO/MXene heterostructures remain unclear, impeding targeted optimization of high-rate performance.

In this study, we propose a controllable solvothermal synthesis method to construct the 0D-2D CoO/MXene vdW heterostructured anode, featuring uniformly anchored CoO nanoparticles on conductive Ti_3_C_2_ MXene nanosheets. The composite exhibits improved interfacial charge transfer, optimized Li⁺ diffusion behavior, and enhanced structural stability, as verified by experimental analysis and DFT calculations. These synergistic effects collectively contribute to exceptionally high-rate performance, making it a promising anode for fast-charging MLIBs. This work establishes a novel and controllable strategy for constructing 0D–2D vdW heterostructures and provides mechanistic insights into structure–property relationships. Moreover, the feasibility in practical MLIBs is validated in a flexible full cell under lithium-limited conditions, offering a pathway toward high-performance anodes and device-level integration.

## Experimental section

### Synthesis of Ti_3_C_2_ MXene nanosheets

Multilayer Ti_3_C_2_ MXene was first prepared by etching 3.0 g of Ti_3_AlC_2_ powder in a solution of 4.8 g LiF in 60 mL of 9 M HCl. The mixture was maintained at 45 °C under magnetic stirring for 36 h until the reaction was complete. The product was washed with deionized water and centrifuged. Delamination was achieved through vortex oscillation. In a typical procedure, the neutral MXene dispersion was oscillated on a vortex shaker at 2300 rpm for 30 min to redisperse the centrifuged precipitate, followed by centrifugation at 10,000 rpm for 5 min. This shaking–centrifugation cycle was repeated twice, after which the dispersion was centrifuged at 3000 rpm for 15 min. The upper black colloidal suspension was collected and freeze-dried to obtain MXene nanosheets.

### Synthesis of CoO nanoparticles

Sixteen mmol of cobalt acetate tetrahydrate was dissolved in 70 mL of ethanol under magnetic stirring. The solution was transferred to a Teflon-lined stainless-steel autoclave and heated at 150 °C for 48 h. After natural cooling to room temperature, the resulting CoO nanomaterial was washed with anhydrous ethanol and vacuum-dried overnight at 80 °C.

### Synthesis of CoO/MXene composites

An appropriate amount of MXene nanosheet powder was dispersed in 70 mL of absolute ethanol and sonicated for 30 min. Subsequently, 16 mmol of cobalt acetate tetrahydrate was added, followed by magnetic stirring to ensure thorough mixing. The mixture was heated at 150 °C for 48 h and cooled naturally to room temperature. The obtained CoO/MXene composites were washed with absolute alcohol, and vacuum-dried overnight at 80 °C. In this study, three CoO/MXene composites with different mass ratios were synthesized by varying the mass of the CoO. Based on ICP-OES analysis, the mass fractions of CoO in the resulting composites, denoted as CoO/MXene-1, CoO/MXene-2, and CoO/MXene-3, were determined to be approximately 68.7%, 62.8%, and 57.7%, respectively.

### Characterizations

The morphology of samples was examined using a field emission scanning electron microscope (FESEM, Hitachi S-4800). Nanostructures and element distributions were investigated via transmission electron microscopy (TEM, Tecnai G2 F30). X-ray diffraction (XRD) patterns were obtained by the MSXD-3 XRD system. Porosity measurements were performed via nitrogen adsorption–desorption isotherms at 77 K using a Micromeritics Tristar 3020 analyzer. Prior to analysis, the samples were degassed under vacuum at 180 °C for at least 6 h. The specific surface area (S_BET_) was calculated using the Brunauer–Emmett–Teller (BET) method in the relative pressure range (P/P_0_) of 0.04–0.20. X-ray photoelectron spectroscopy (XPS) was conducted on an X-ray photoelectron spectrometer (Thermo Escalab 250Xi) with Al Kα radiation (hν = 1486.6 eV). All binding energies were calibrated using the carbon contaminant (C 1 s = 284.6 eV). The Co and Ti contents in the composites were determined by inductively coupled plasma optical emission spectrometry (ICP-OES, Agilent ICPOES730).

### Electrochemical measurements

Electrochemical measurements were performed by assembling Swagelok cells within an argon-filled glove box. The active material, acetylene black (Super P), and polyvinylidene fluoride (PVDF) were homogeneously mixed and ground together at a mass ratio of 7:2:1. A suitable quantity of *N*-methyl-2-pyrrolidone (NMP) was introduced to the mixture, and thorough mixing was achieved using a planetary mixer (THINKY MIXER ARE-310) to form a uniform slurry. This slurry was subsequently applied to copper foil via a four-roll coater, and then subjected to vacuum drying at 80 °C for 12 h to fabricate the electrode sheets. Electrodes were punched from the dried electrode sheet, with an average active material loading of ~1.2 mg cm^−^^2^. The electrodes were separated by Celgard separators, and lithium foil was used as both the counter and reference electrode. The electrolyte was 1.0 M LiPF_6_ in 1:1:1 (by volume) ethylene carbonate (EC)/dimethyl carbonate (DMC)/ethyl methyl carbonate (EMC). The cyclic charge and discharge tests of the coin cells were conducted using a LAND CT2001A battery testing system, and the voltage range was set from 0.01 to 3.0 V (vs Li/Li^+^). Electrochemical analyses, including cyclic voltammetry (CV) test from 0.01 to 3.0 V at a scan rate of 0.2 mV s^−^^1^ and electrochemical impedance spectroscopy (EIS) test ranging from 10^−^^2^ to 10^6^ Hz, were conducted using an AUTOLAB electrochemical workstation.

### First principles calculations

All calculations were performed within the framework of DFT using the Vienna Ab initio Simulation Package (VASP 6.3.0). The generalized gradient approximation (GGA) of the Perdew–Burke–Ernzerhof (PBE) function was used to describe the exchange-correlation energy. Interactions between valence electrons and ionic cores were described using the projector augmented wave (PAW) method and pseudopotentials. A 3 × 3 × 3 k-point grid under Monkhorst-Pack is used in the optimization process, and the truncation energy of 450 eV was employed. Full structural relaxations were carried out for both lattice parameters and atomic positions, with energy and force convergence thresholds set to 10^−6 ^eV and 0.03 eV/Å, respectively. To account for the localization effect of *d*-orbital electrons in transition metal atoms, the Hubbard+U method was applied with effective *U*-values of 2.5 eV for both Co and Ti.

## Results and discussion

**Fig. 1:**
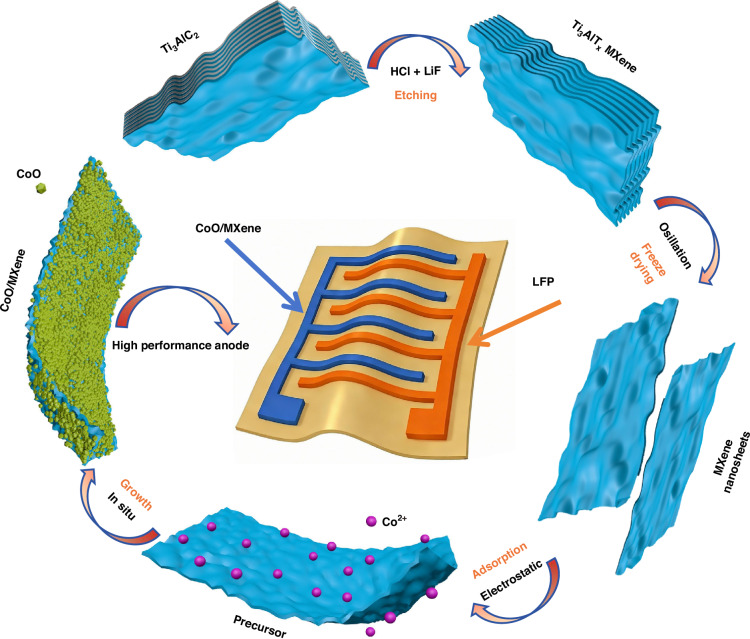
Schematic illustration of the fabrication process for the CoO/MXene composite

The fabrication process of CoO/MXene 0D-2D vdW heterostructure is demonstrated in Fig. 1. Multilayer Ti_3_C_2_ MXene was synthesized from Ti_3_AlC_2_ (MAX phase) by selective etching of Ti-Al metallic bonds using HCl and LiF^33^. The obtained multilayer Ti_3_C_2_ MXene was subsequently delaminated via vortex oscillation and then freeze-dried to obtain MXene nanosheet powder^34^. MXene nanosheet powder was ultrasonically dispersed in ethanol, followed by the addition of cobalt acetate tetrahydrate (Co(Ac)_2_·4H_2_O) under vigorous magnetic stirring. Owing to the abundant surface functional groups, negatively charged MXene nanosheets effectively adsorbed Co^2+^ cations through electrostatic interactions, forming the Co/MXene precursor^35,36^. Notably, ethanol was used as the reaction solvent to suppress oxygen participation, thereby minimizing the oxidation of MXene. Acting as a 2D conductive matrix, MXene facilitates fast and reversible electron and ion transport, mitigates CoO nanoparticle agglomeration during lithiation/delithiation, and alleviates its volume-related effects at the interface. Additionally, CoO nanostructures provide shortened lithium-ion diffusion pathways and additional active sites. By adjusting the mass ratio of the MXene nanosheet powder to the cobalt precursor, the loading density of CoO on MXene could be effectively tuned. Three CoO/MXene composites with different mass ratios were prepared and subjected to identical electrochemical testing conditions.

**Fig. 2:**
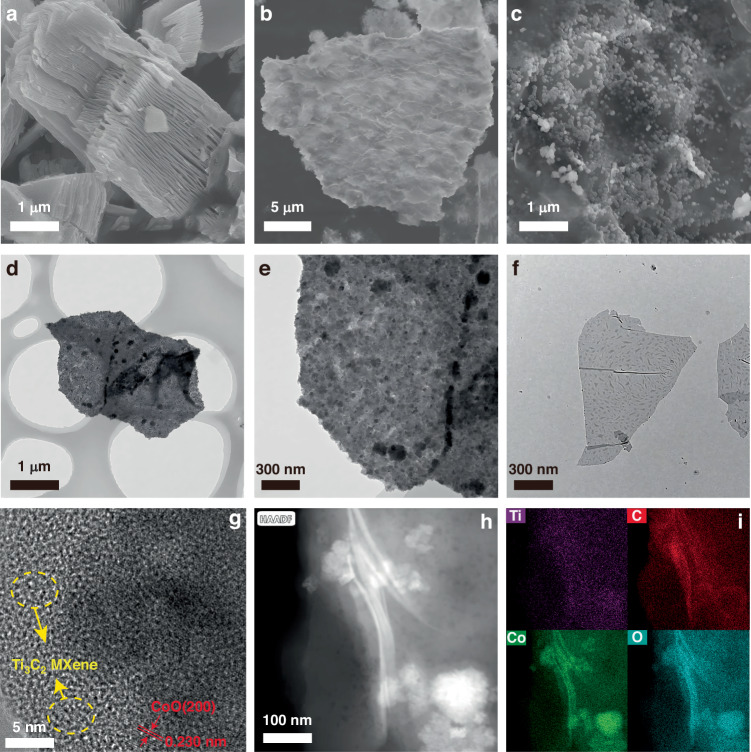
SEM images of **a** multilayer Ti_3_C_2_ MXene, **b**, **c** CoO/MXene composites, TEM images of **d**, **e** CoO/MXene composite, **f** MXene nanosheets, **g** HRTEM image, and **h, i** Scanning TEM images and elemental mapping images of CoO/MXene composites

As shown in Fig. 2a, the in-situ wet chemical etching process effectively removed the Al layers from the Ti_3_AlC_2_ MAX phase, while preserving its inherent layered structure. Subsequent delamination was achieved through vortex oscillation, resulting in the production of MXene nanosheet powder following freeze-drying. The TEM image in Fig. 2f confirms that multilayer MXene was successfully exfoliated into nanosheets with larger lateral dimensions and fewer structural defects. Pristine CoO nanoparticles, as shown in Fig. S1, exhibit smooth surfaces with particle sizes ranging from 200 to 800 nm. By varying the mass of the MXene nanosheet powder, CoO/MXene composites with different mass ratios were synthesized. Their elemental compositions were analyzed using inductively coupled plasma spectroscopy, as illustrated in Fig. S2. Among the samples, CoO/MXene-1 exhibited the highest cobalt content, resulting in a dense distribution of CoO nanoparticles. However, a fair number of particles fail to adhere to the surface of MXene nanosheets (Fig. S3a, b). In contrast, CoO/MXene-3, which has the lowest cobalt content, showed a more dispersed distribution of CoO across the MXene nanosheets, thus underutilizing the available active sites (Fig. S3e, f). In the case of CoO/MXene-2, with an intermediate cobalt content, CoO nanoparticles were uniformly and densely distributed (Fig. S3c, d). Figure 2b reveals a large 2D sheet morphology with prominent surface wrinkles, contributing to the high-temperature reaction. At higher magnification (Fig. 2c), the MXene nanosheet surfaces are decorated with abundant CoO nanoparticles, some of which are even partially embedded within the sheets. This observation demonstrates the formation of a 0D-2D vdW heterostructure. Such a unique architecture enhances the structural integrity of the composite, establishes a fast electron transport network, and mitigates CoO nanoparticle aggregation and volume expansion during lithiation/delithiation. TEM results in Fig. 2d, e further validate this structure, showing CoO nanoparticles distributed on the MXene surface, in contrast to the bare MXene nanosheet in Fig. 2f. These results confirm that MXene provides abundant nucleation sites for the in-situ growth of CoO. Figure 2g demonstrates a lattice spacing of 0.23 nm, corresponding to the (200) crystal plane of CoO (JCPDS 43-1004)^23^. Moreover, Fig. 2h, i further reveal the 2D structure and the coexistence of Ti, C, Co, and O in the composites, which provides additional evidence for the successful formation of the 0D-2D CoO/MXene vdW heterostructure.

**Fig. 3:**
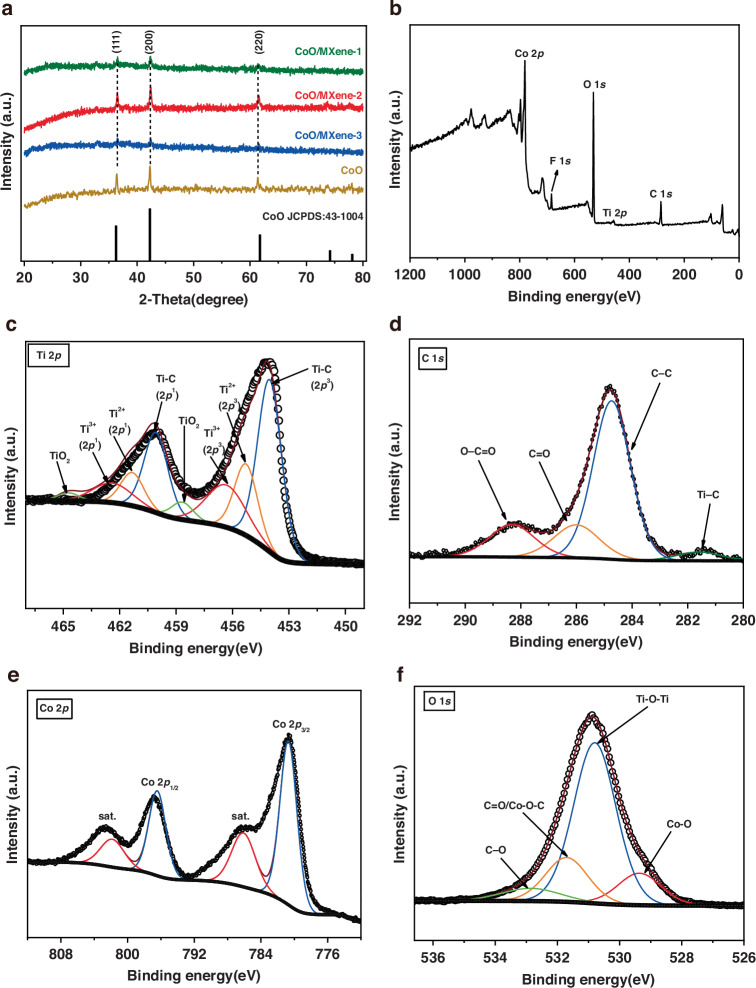
**a** XRD pattern of CoO and CoO/MXene composites. **b** XPS survey spectrum of CoO/MXene composites. High-resolution XPS spectra of CoO/MXene composites **c** Ti 2*p*, **d** C 1*s*, **e** Co 2*p*, and **f** O 1*s*

The XRD pattern of Ti_3_AlC_2_ is in perfect agreement with the card (JCPDS 52-0875) and lacks any impurity peaks (Fig. S4), indicating a high purity of the MAX phase^37^. After etching and delamination, most diffraction peaks of Ti_3_AlC_2_ disappear, and the original narrow peak at 9.5° broadens and shifts to a lower angle, corresponding to the (002) plane of MXene nanosheets^38^. Importantly, no characteristic peaks of TiO_2_ are observed, indicating that the MXene was not oxidized during the synthesis process^39^. Figure 3a shows XRD patterns of CoO and the three CoO/MXene 0D-2D vdW heterostructures, all displaying similar features. The three diffraction peaks at 36.5°, 42.4°, and 61.5° are attributed to the (111), (200), and (220) crystal planes of CoO (JCPDS 43-1004), respectively^40^. Notably, no peaks associated with rutile-type TiO_2_ are observed, further confirming that the MXene component remains unoxidized in all three composites. Additionally, nitrogen adsorption-desorption isotherms were examined to characterize surface area and pore properties. As depicted in Fig. S5, the isotherms for CoO/MXene display a typical H3 hysteresis loop with type IV isotherms, indicative of a mesoporous structure. The S_BET_, calculated using the BET method, is 59.86 m^2^ g^−^^1^, suggesting a large surface area that provides abundant active sites for lithium-ion storage and facilitates efficient interfacial charge transfer. Furthermore, the average pore size distribution curve (inset of Fig. S4) indicates mesopores predominantly ranging from 2 to 24 nm.

Compared with the XPS spectrum of MXene nanosheets (Fig. S6a), CoO/MXene composites reveal the presence of Co and O elements from CoO, alongside Ti, C, and F elements from MXene nanosheets (Fig. 3b). For the high-resolution Ti 2*p* spectrum of CoO/MXene composites (Fig. 3c), the peaks at 453.7, 455.0, and 457.3 eV correspond to Ti-C (sp^3^), Ti^2+^ (sp^3^), and Ti^3+^ (sp^3^)^41^, respectively, consistent with those observed for MXene nanosheets (Fig. S6b). This confirms that no significant oxidation of MXene occurred during composite synthesis. The C 1s spectrum of the composite (Fig. 3d), peaks at 286.0 eV and 288.3 eV correspond to C=O and O–C=O bonds, respectively^40^. And the peak at 288.9 eV in the MXene nanosheets arises from C–F bonds introduced during the in-situ etching process (Fig. S6c). The Co 2*p* spectrum (Fig. 3e) exhibits two characteristic peaks at 780.7 and 796.4 eV, attributed to Co 2*p*_3/2_ and Co 2*p*_1/2_ spin-orbit splitting, respectively, in agreement with previously reported XPS data for CoO nanomaterials^42,43^. Compared with MXene nanosheets (Fig. S6d), the deconvolution of the O 1s spectrum for the CoO/MXene composite (Fig. 3f) reveals three primary components. The peak at ~529.7 eV is attributed to the lattice oxygen (O^2^^−^) in CoO. The peak at ~531.3 eV originates from the oxygen-containing functional groups (–O, –OH) on the MXene surface. An additional minor peak at ~533 eV corresponds to adsorbed molecular water or C-OH/C-O-C species. Critically, no distinct peak assignable to a new interfacial chemical bond (e.g., Ti–O–Co) is observed, supporting the inference of a van der Waals-dominated heterointerface^44^. Furthermore, the solvothermal strategy demonstrated here is not limited to MXene. The key factors enabling the successful formation of the CoO/MXene heterostructure are the abundant surface functional groups (–O, –OH, –F) and the negatively charged surface of MXene, which together promote electrostatic adsorption of Co^2^⁺ ions and subsequent in situ nucleation. This principle suggests that the approach can be extended to other 2D materials, provided they possess sufficient surface functionalities or can be chemically modified to introduce negatively charged sites.

**Fig. 4:**
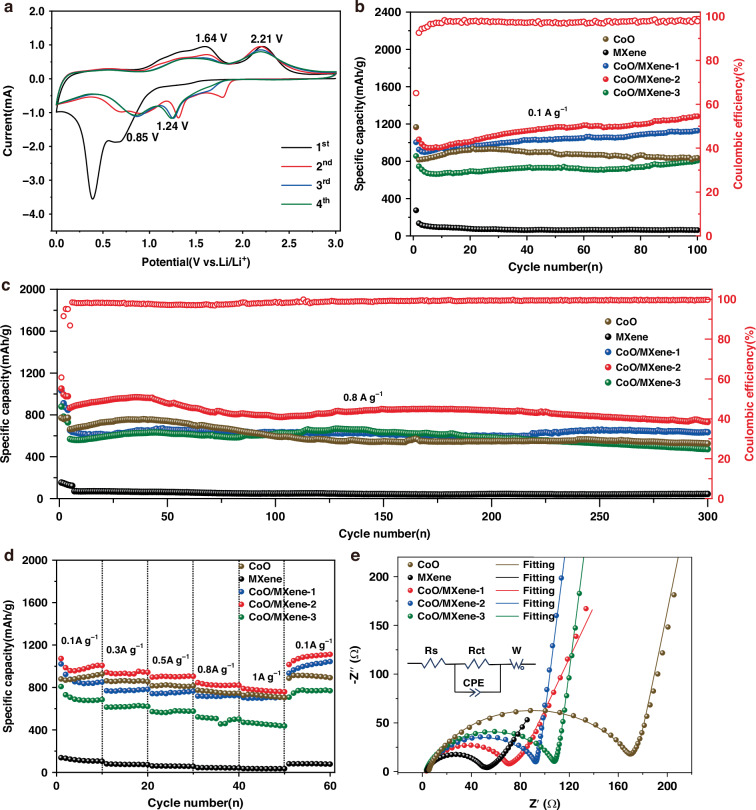
**a** CV curves of CoO/MXene electrodes at a scan rate of 0.2 mV s^−1^. **b** Cycling performance at 0.1 A g^−1^. **c** Long cycle performance at 0.8 A g^−1^ for 300 cycles. **d** Rate capabilities and **e** Nyquist plots of CoO/MXene in comparison to CoO and MXene

The electrochemical performance was systematically evaluated. Figure 4a illustrates the initial four CV plots of CoO/MXene-2 under 0.2 mV s^−^^1^ within a voltage window of 0.01 ~ 3.0 V. In the first cathodic scan, a reduction peak appears at 0.39 V but disappears in subsequent cycles. This peak is attributed to the initial conversion reaction during Li⁺ insertion into CoO (CoO + 2Li⁺ + 2e^−^ → Co + Li_2_O), along with irreversible reactions between the electrolyte and the electrode surface leading to the formation of a stable solid electrolyte interphase (SEI) layer^45^. In the following cycles, two reduction peaks around 0.85 V and 1.24 V are associated with the electrochemical reduction reactions of CoO via lithium intercalation.^46^ On the anodic sweep, two oxidation peaks around 1.64 V and 2.21 V correspond to the re-oxidation of Co to CoO, accompanied by lithium deintercalation (Co + Li_2_O → CoO + 2Li^+^ + 2e^−^)^47^. In the subsequent scans, the near-overlapping positions of the oxidation peaks and reduction peaks indicate excellent electrochemical reversibility and cycling stability of the CoO/MXene electrode.

The cycling ability of CoO/MXene composites was further assessed, as shown in Fig. 4b. During a constant current of 0.1 A g^−^^1^, the CoO/MXene-2 electrode demonstrates the highest initial capacity of 1163.9 mA h g^−^^1^ with a coulombic efficiency of 65.1%. After 100 cycles, it retains a high reversible capacity of 1282.3 mAh g^−^^1^, outperforming the CoO (836.1 mAh g^−^^1^), CoO/MXene-1 (1127.8 mAh g^−^^1^), and CoO/MXene-3 (806.5 mAh g^−^^1^) electrodes. The gradual capacity increase during cycling is attributed to multiple reversible mechanisms: (i) additional Li⁺ storage via reversible formation/decomposition of a polymeric gel-like layer, which is widely recognized in conversion-type electrodes^48–50^; (ii) the conversion of CoO generates metallic Co nanoparticles embedded in a Li_2_O matrix, creating abundant Li_2_O/Co heterointerfaces and accommodate additional lithium through a capacitive-type interfacial storage^51,52^; and (iii) continuous electrochemical activation through slight particle pulverization and exposure of new active surfaces, which enlarge the electrochemically active surface area and improve Li⁺ accessibility, leading to enhanced capacity over extended cycling^16,53^. The initial low Coulombic efficiency is due to irreversible SEI formation, while the subsequent capacity rise is dominated by highly reversible processes, as evidenced by the stable efficiency exceeding 98.5% after initial cycles. Further long-term cycling tests at 0.8 A g^−^^1^ were conducted for the three composite electrodes (Fig. 4c). After 300 cycles, the CoO/MXene-2 electrode exhibited a consistently high reversible capacity of 731.7 mA h g^−^^1^, attributed to the uniform dispersion of CoO nanoparticles on the MXene nanosheets. This 0D-2D vdW heterogeneous nanostructures effectively mitigates volume expansion and prevents electrode pulverization during lithiation/delithiation. Moreover, at a higher current density of 1 A g^−^^1^, the CoO/MXene-2 electrode still delivered excellent performance, maintaining a reversible capacity of 500.6 mAh g^−^^1^ with a capacity retention of 78.6% after 500 cycles (Fig. S7). In contrast, the CoO electrode exhibited severe capacity degradation, remaining only 279.4 mAh g^−^^1^ due to its poor conductivity and structural instability during rapid charge-discharge cycles. These results demonstrate that the 0D-2D vdW heterostructure significantly enhances both ion and electron transport, leading to improved structural stability and superior cycling performance. It is noted that the cycle numbers were selected based on specific evaluation goals: 100 cycles at the low rate (0.1 A g^−^^1^) are sufficient to capture the electrochemical activation and verify the intrinsic capacity, whereas extended cycling (300 cycles) at the high rate (0.8 A g^−^^1^) is required to rigorously assess the long-term mechanical stability under high-rate stress.

Rate capability tests were performed to assess the electrodes’ kinetic behavior. The current density was modulated in ten-cycle intervals, increasing from 0.1 A g^−^^1^ to 1 A g^−^^1^, and then returning to 0.1 A g^−^^1^. The reversible capacities of the five electrodes are summarized in Table S1. Among them, the CoO/MXene-2 composite electrode obtain reversible capacities of 1006.6, 943.6, 904.6, 820.7, and 759.2 mA h g^−^^1^ at current densities of 0.1, 0.3, 0.5, 0.8 and 1 A g^−^^1^, respectively, outperforming the other electrodes (Fig. 4d). After the current density was reduced from 1 A g^−^^1^ back to 0.1 A g^−^^1^, the reversible capacity of the CoO/MXene-2 electrode recovered to 1111.6 mA h g^−^^1^ after ten cycles. This significant increase in capacity is attributed to the gradual activation of electrode active materials, ultimately surpassing the initial reversible capacity. These results confirm the excellent rate capability and fast charge/discharge characteristics of the CoO/MXene 0D-2D vdW heterostructure.

EIS measurements were conducted to investigate the charge transport behavior. As shown in Fig. 4e, the Nyquist plots of all electrodes were fitted using the equivalent circuit shown in the inset, and the fitting parameters are listed in Table S2. The CoO/MXene-2 electrode exhibited the lowest charge transfer resistance (*R*_ct_ = 60.77 Ω), indicating the fastest electron transport and thus superior cycling stability. The lithium storage performance of various CoO-based composite anodes is summarized in Table S3, highlighting the outstanding electrochemical performance of the CoO/MXene composite. Notably, while most counterparts operate at low current densities (0.1–0.2 A g^−^^1^), our anode sustains a robust capacity of 731.7 mAh g^−^^1^ even at a significantly higher rate of 0.8 A g^−^^1^, surpassing previous CoO/MXene composites and binary oxides. Although some multi-component carbon-based anodes exhibit higher absolute capacities at lower rates, our design achieves a superior balance of high-rate capability and long-term stability, which is pivotal for high-rate micro-batteries.

**Fig. 5:**
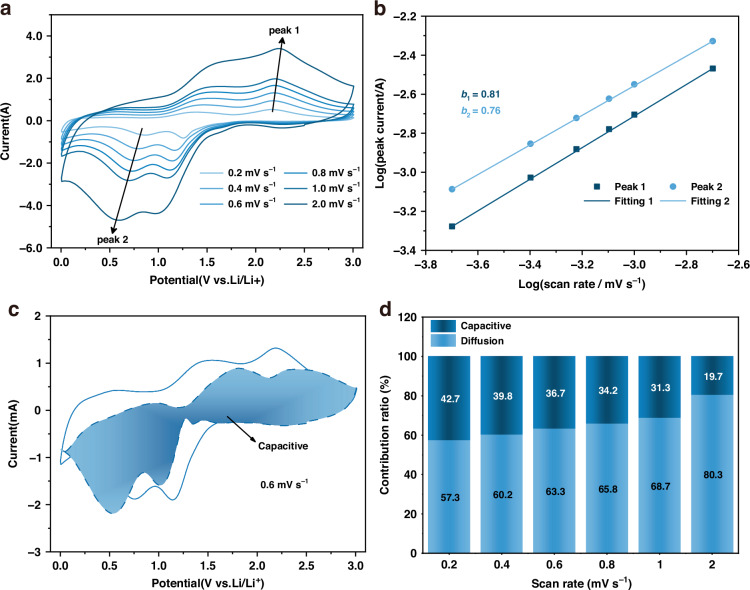
**a** CV curves of the CoO/MXene-2 electrode at various scan rates from 0.2 to 2 mV s^−1^. **b** Relationship between the log (peak current) and log (scan rate). **c** Contribution of capacitive charge storage to the total capacity of the CoO/MXene-2 electrode at a scan rate of 0.6 mV s^−1^. **d** Contribution ratio of the capacitive and diffusion-controlled charge storage at different scan rates

Ti_3_C_2_ MXene is typically considered a pseudocapacitive material due to its surface redox activity. To elucidate the lithium storage mechanism of the CoO/MXene composites, CV tests were performed on the CoO/MXene-2 electrode at scan rates ranging from 0.2 to 2 mV s^−1^. As shown in Fig. 5a, the CV curves retain similar shapes but gradually broaden with increasing scan rate. The lithium storage mechanisms generally involve diffusion and capacitive contributions. In this case, the peak current does not scale linearly with the square root of the scan rate, suggesting a mixed kinetic behavior involving both mechanisms^47^. A more quantitative understanding can be achieved by analyzing the current–scan rate relationship, as described by the following equation:

1 $${\rm{i}}={a}{{\rm{\nu}}^{b}}$$

where, *a* and *b* are fitting constants, and the *b* value is associated with charge storage behavior. A *b* value of 0.5 indicates a diffusion-controlled process, whereas a value of 1.0 suggests a capacitive-controlled process^54^. For the CoO/MXene electrode, the fitted values of *b* were 0.81 and 0.76, implying a hybrid mechanism involving both diffusion and capacitive contributions (Fig. 5b).

To quantitatively separate these contributions, the current response (*i*) was expressed as the sum of capacitive-controlled (*k*_1_ν) and diffusion-controlled (*k*_2_ν^0.5^) processes:

2 $${\rm{i}}={\rm{k}{1}{\rm{\nu}}}+{\rm{k}{2}{\rm{\nu}}}^{0.5}$$

where *k*_1_ and *k*_2_ are constants derived from the scan rate^55^. At a scan rate of 0.6 mV s^−1^, capacitive contributions accounted for 63.3% (Fig. 5c). As illustrated in Fig. 5d, the capacitive contribution increases progressively with scan rate, further confirming the dominance of pseudocapacitive behavior in the CoO/MXene electrode. This pseudocapacitive effect is beneficial for fast charge/discharge capability.

**Fig. 6:**
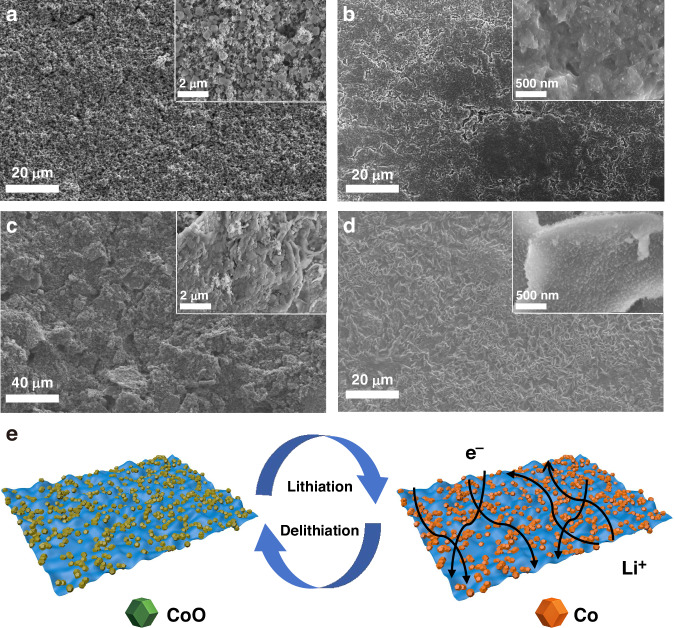
Morphology images of the CoO electrode before (**a**) and after (**b**) 300 cycles at 0.8 A g^−1^. Morphology images of CoO/MXene-2 electrode before (**c**) and after (**d**) 300 cycles at 0.8 A g^−1^. **e** Schematic illustration of the advantages of MXene for improved lithium storage of CoO

The morphological evolution of the CoO and CoO/MXene-2 electrodes before and after cycling was investigated by SEM to further validate the structural stability. As shown in Fig. 6a, b, the pristine CoO electrode comprises well-dispersed nanoparticles, which aggregate into larger nanocrystalline clusters accompanied by prominent surface cracks after prolonged cycling. This degradation arises from severe volume expansion and contraction, stemming from CoO’s inherently poor conductivity and low lithium-ion diffusion coefficient. In contrast, when CoO is integrated with MXene nanosheets to form a 0D-2D vdW heterostructure, the resulting CoO/MXene-2 electrode exhibits markedly enhanced structural integrity. As shown in Fig. 6c, d, the CoO/MXene-2 composite retains its initial heterostructure morphology with no visible surface cracks even after extensive cycling, indicating that the MXene matrix effectively mitigated the volumetric effects of CoO during lithiation/delithiation. Figure 6e illustrates the advantages of the heterostructure: the highly conductive MXene sheets facilitate fast electron and ion transport, while their flexible 2D layers buffer the mechanical stress induced by CoO expansion. Furthermore, the strong interfacial interactions within the 0D-2D heterostructure securely anchor CoO nanoparticles onto the MXene layers, preventing their detachment during repeated cycling.

**Fig. 7:**
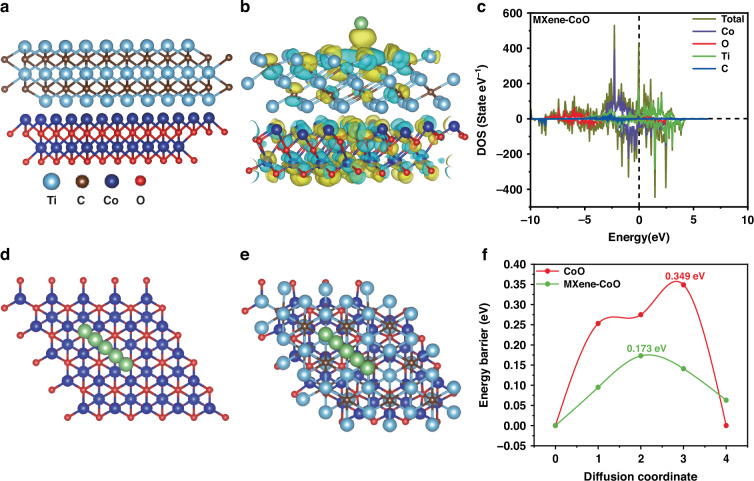
First-principles calculation. **a** MXene-CoO heterostructure. **b** Differential charge density after Li insertion of MXene-CoO heterostructure. **c** Density of states of MXene-CoO heterostructure. Li diffusion pathways on **d** CoO surface and **e** MXene-CoO surface. **f** Diffusion barriers of Li on CoO and CoO-MXene surfaces

To elucidate the enhancement mechanism of the MXene-CoO heterostructure in electrochemical performance, we performed first-principles calculations based on DFT to investigate its electronic structure and lithium adsorption behavior. The structural model used in the calculations is shown in Fig. 7a. The Ti_3_C_2_ MXene model employed in this DFT study is constructed without surface functional groups (–F, –OH, –O) to form an idealized structure. This simplification is adopted to primarily investigate the intrinsic electrochemical properties of the Ti_3_C_2_ matrix, such as its inherent metallic conductivity, layered Li⁺ diffusion pathways, and the activity of Ti sites, without the complicating effects of functional terminations^56–59^. While this approach provides fundamental insights, we acknowledge that it does not fully capture the initial surface chemistry of experimental MXene. Thus, our results should be interpreted as revealing the intrinsic behavior of the Ti_3_C_2_ framework, with functionalized models reserved for future in-depth studies.

### Differential charge density

To probe the interfacial charge transfer mechanism during lithium atom adsorption, the electron redistribution was visualized through differential charge density analysis^60^. The differential charge density distributions of a lithium atom adsorbed on the CoO (Fig. S8) and MXene-CoO (Fig. 7b) surfaces were calculated, where yellow and blue regions indicate electron accumulation (charge enrichment) and depletion (charge deficiency), respectively. Lithium atom adsorption on the CoO surface leads to electron accumulation primarily above cobalt (Co) atoms, indicating notable Li–Co interactions. In contrast, in the MXene-CoO system, the electron cloud is mainly concentrated above the titanium (Ti) atoms, forming a strong localized electron distribution between the lithium atom and the material surface. The enhanced charge coupling effect suggests that lithium has a stronger chemical adsorption on the MXene-CoO. Compared to pristine CoO, the MXene-CoO heterostructure significantly improves the charge transfer capability between lithium atoms and the substrate, inducing stronger ionic interactions. Such strong interactions improve the lithium atom adsorption stability, inhibit aggregation, and prevent lithium cluster formation, thereby increasing cycling stability and specific capacity^61^.

### Density of state

To understand how MXene incorporation affects the electronic conductivity of CoO, we calculated the density of states (DOS) to evaluate the electronic structure. Figure 7c shows the DOS plot for the MXene-CoO heterostructure, where the Fermi level is set to 0 eV (black dashed line). The total DOS is represented by a dark green line, while the projected density of states (PDOS) for cobalt (Co), oxygen (O), titanium (Ti), and carbon (C) atoms are represented by purple, red, green, and blue lines, respectively. Compared to pristine CoO (Fig. S9), the MXene-CoO heterostructure exhibits a significantly higher DOS at the Fermi level, indicating enhanced metallicity and improved electrical conductivity. Analyzing the distribution intervals of the DOS, the peak values for CoO are primarily concentrated within the ranges of −9 to −4 eV and −3 to 2 eV, with a relatively narrow distribution. In contrast, the MXene-CoO heterostructure exhibits a broader DOS distribution (from −10 to 4 eV) with delocalized peaks, indicating enhanced electronic state hybridization with occupied states covering a broader energy range. This complex electronic state distribution provides additional active sites and pathways for electron transfer, thereby facilitating chemical reactions^62^. The introduction of MXene significantly increases DOS near the Fermi level, primarily attributed to the substantial contribution of Ti atoms (green line). This indicates a large number of available electron states for occupation, suggesting that the MXene incorporation effectively optimizes the electronic structure, thereby significantly enhancing its electrical conductivity^63^.

### Diffusion properties on MXene-CoO heterostructure

The migration rate of lithium atoms is a critical factor in evaluating the rate capability of anode materials, and is primarily determined by the diffusion energy barrier between adjacent stable adsorption sites^64,65^. Specifically, a lower diffusion barrier indicates faster Li^+^ transport, improving charging/discharging rates and overall battery performance^66^. The climbing image nudged elastic band (CI-NEB) simulations are employed to calculate the Li⁺ migration pathways and corresponding diffusion barriers of CoO (Fig. 7d) and MXene (Fig. 7e), with results summarized in Fig. 7f. The calculated migration barrier decreases dramatically from 0.349 eV in CoO to 0.173 eV in MXene-CoO heterostructure, indicating that MXene incorporation facilitates fast Li⁺ migration^67^.

Therefore, the formation of the MXene-CoO heterostructure improves electronic conductivity by increasing the DOS near the Fermi level, strengthening interfacial lithium adsorption via optimized charge transfer dynamics, and reducing the diffusion energy barrier to accelerate Li^+^ transport. This synergistic mechanism facilitates rapid ion diffusion and stable interfaces, making MXene-CoO a promising anode material for advanced high-rate, long-life lithium-ion batteries.

**Fig. 8:**
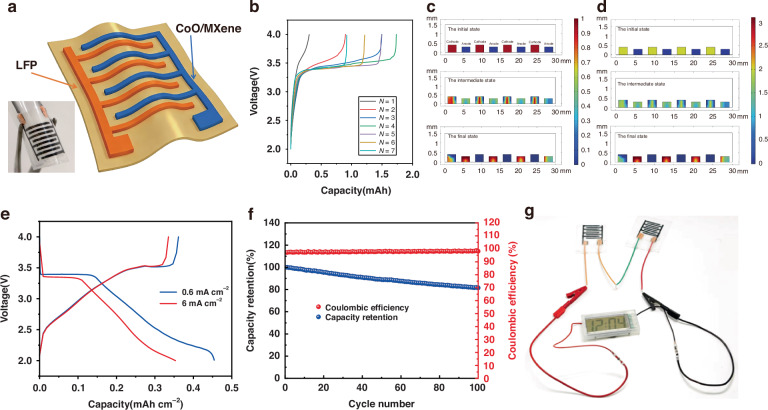
Design optimization, performance, and application of the flexible CoO/MXene-based micro full cell. **a** Schematic illustration of the flexible interdigitated full-cell device; the inset shows a photograph of the actual encapsulated device.**b**–**d** Finite element simulation results of the full cell: **b** calculated capacity for different numbers of interdigitated finger pairs (N), and simulated spatial distributions of **c** state of charge and **d** Li⁺ concentration. **e**, **f** Experimental electrochemical performance of the fabricated full cell: **e** typical galvanostatic charge–discharge voltage profiles and **f** cycling performance. **g** Application demonstration of the flexible full cell powering a digital clock

To demonstrate the practical viability of the CoO/MXene anode in practical micro energy storage systems, a flexible full battery was fabricated using inkjet printing. Distinct from half-cell configurations that rely on an infinite lithium source (lithium foil), this device was assembled with a lithium iron phosphate (LFP) cathode to evaluate performance under limited lithium inventory conditions. This setup more accurately reflects the constraints of practical micro-batteries. As explicitly illustrated in the schematic in Fig. 8a, both the CoO/MXene anode and LFP cathode were printed onto a flexible substrate in an interdigitated architecture to optimize ion transport paths. The device was then activated with the electrolyte and encapsulated within a flexible polyethylene layer to ensure mechanical integrity and environmental isolation. The inset in Fig. 8a displays an optical photograph of the actual encapsulated device. The electrochemical performance of the assembled full cell was evaluated by galvanostatic charge/discharge cycling within a voltage window of 2.0–4.0 V.

To elucidate the internal electrochemical dynamics and optimize the device architecture, a finite element model of the interdigitated full-cell was established using COMSOL Multiphysics. The spatial-temporal evolution of key parameters, including cell voltage, lithium-ion concentration, and state of charge (SOC), was simulated to characterize the charge–discharge behavior. Based on these simulations, a capacity analysis was conducted to guide the structural design of the interdigitated electrodes. The effect of the number of interdigitated finger pairs (N) was examined under a fixed total device area. As shown in Fig. 8b, the voltage–capacity curves for different N indicate that the maximum capacity is achieved at *N* = 4. This optimum arises from a fundamental trade-off: increasing N shortens the ion diffusion distance and enhances the electrochemical kinetics, whereas an excessively large N increases the volume fraction of non-active gaps, reducing the active material loading and achievable capacity. The corresponding spatial distributions of SOC, lithium-ion concentration, and cell voltage at the initial, intermediate, and final charging stages are presented in Figs. 8c, d and S10. These results provide physical insight into the structure–performance relationship, offering theoretical guidance for the rational design of the battery architecture.

The electrochemical performance of the practical full-cell device was further evaluated. Figure 8e displays the typical galvanostatic charge–discharge voltage characteristics. The full cell delivers the areal capacity of 0.45 mAh cm^−^^2^ at a high current density of 6 mA cm^−^^2^. The long-term cycling stability is presented in Fig. 8f, revealing a capacity retention of 81.5% after 100 cycles. These results demonstrate the excellent areal capacity and cyclability of the device in a full-cell configuration, corroborating the structural advantages identified in the simulations. As benchmarked in Table S4, the device demonstrates exceptional competitiveness in areal capacity under high-rate conditions compared to state-of-the-art counterparts. Furthermore, a functional demonstration was conducted to validate the system-level integration capability. As shown in Fig. 8g, the assembled flexible full cells directly power a commercial digital clock and sustain its continuous operation. This successful demonstration confirms that the MLIB based on the CoO/MXene interdigitated electrodes can function effectively as a robust and complete micro-power system for wearable and miniaturized electronic applications.

Although the prototype device demonstrated in this work exhibits promising electrochemical performance, it should be noted that the use of a conventional liquid electrolyte remains a key limitation for practical MLIBs in terms of long-term encapsulation reliability, safety, and integration compatibility. The choice of a liquid system at this stage serves to clearly evaluate the intrinsic performance of the electrode materials. To address these limitations toward practical MLIB integration, future work will focus on developing solid-state configurations by depositing LiPON solid electrolyte layers via magnetron sputtering, aiming to improve encapsulation robustness, eliminate leakage risks, and enhance compatibility with thin-film and on-chip processing.

## Conclusion

This study presents a facile solvothermal strategy for constructing 0D-2D vdW CoO/MXene heterostructures, enabling the controlled growth and stacking of CoO nanoparticles on conductive Ti_3_C_2_ nanosheets. The strong synergistic effect between CoO and MXene imparts the composite with excellent lithium storage performance, achieving a reversible capacity of 731.7 mA h g^−^^1^ after 300 cycles by combining the high capacity of CoO with the conductivity and stability of MXene. Notably, the proposed design strategy is generalizable and can be extended to other TMOs (e.g., Fe_2_O_3_, MnO, NiO) and various 2D materials (e.g., graphene, TMDs, MBene) for applications in sodium-ion batteries and supercapacitors. Despite these advances, certain limitations remain, including the scalability of the synthesis process due to its complexity and energy consumption, as well as the long-term stability of the heterostructure under practical operating conditions. Future efforts will focus on optimizing synthesis toward improved scalability, exploring sustainable functionalized MXene substrates, and employing in situ/operando techniques to gain deeper insights into interfacial ion/electron transport. Overall, this study establishes a controllable 0D–2D vdW heterostructure design strategy and a material–mechanism–device validation framework, offering experimental and theoretical insights into structure–property relationships and providing a practical pathway toward high-performance micro energy storage devices.

## Supplementary information

Revised_Supplementary material
