# Supplementary material for: A synergistic CoO/MXene heterostructure anode with facilitated interfacial charge transfer for high-rate micro lithium-ion batteries
Source: Microsyst Nanoeng. 2026 May 12;12:172. doi: 10.1038/s41378-026-01246-9 (PMC13168570; doi:10.1038/s41378-026-01246-9)
Supplement: Supplementary file 1 — Revised_Supplementary material [file 41378_2026_1246_MOESM1_ESM.pdf]

A Synergistic CoO/MXene Heterostructure Anode with Facilitated Interfacial Charge Transfer for High-Rate Micro Lithium-Ion Batteries

Bingmeng Hu [a, b], Hanjing Wei [a], Hui Zhou [a], Titao Fang [a], Hailong Wang [a], Shixin Wang [a], Jinyang Han [b], Chenpeng Huang [d], Xiaoming Zhang [a, c*], Xiaohong Wang [e*]

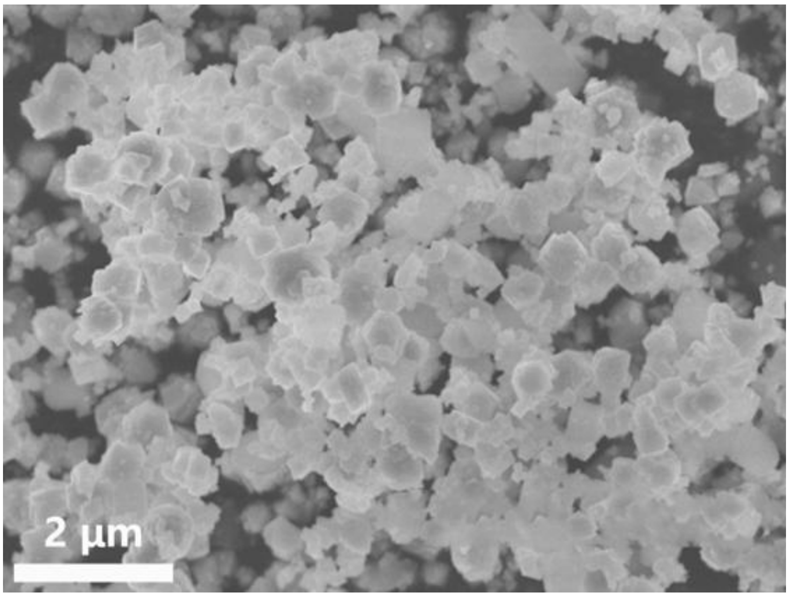

Figure S1 SEM image of pure CoO nanoparticles.

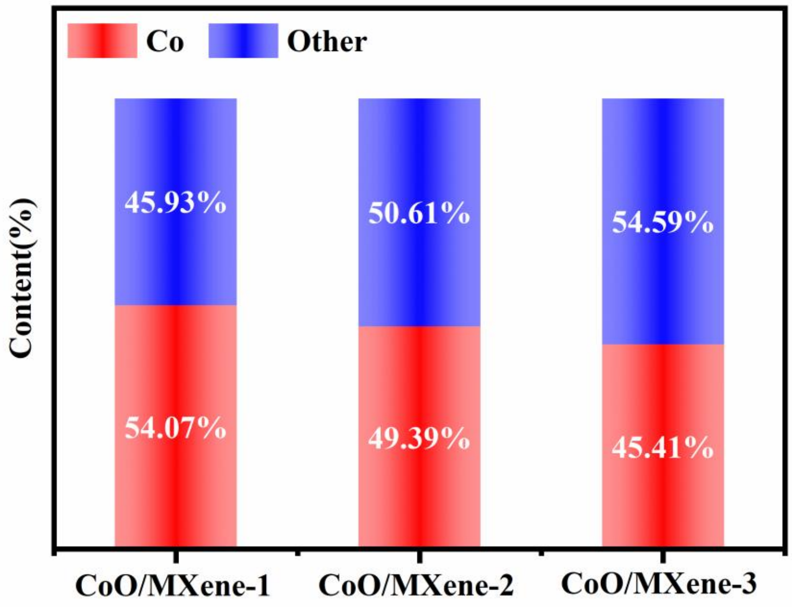

Figure S2 Mass proportion diagram of Co element in CoO/MXene composites with different proportions.

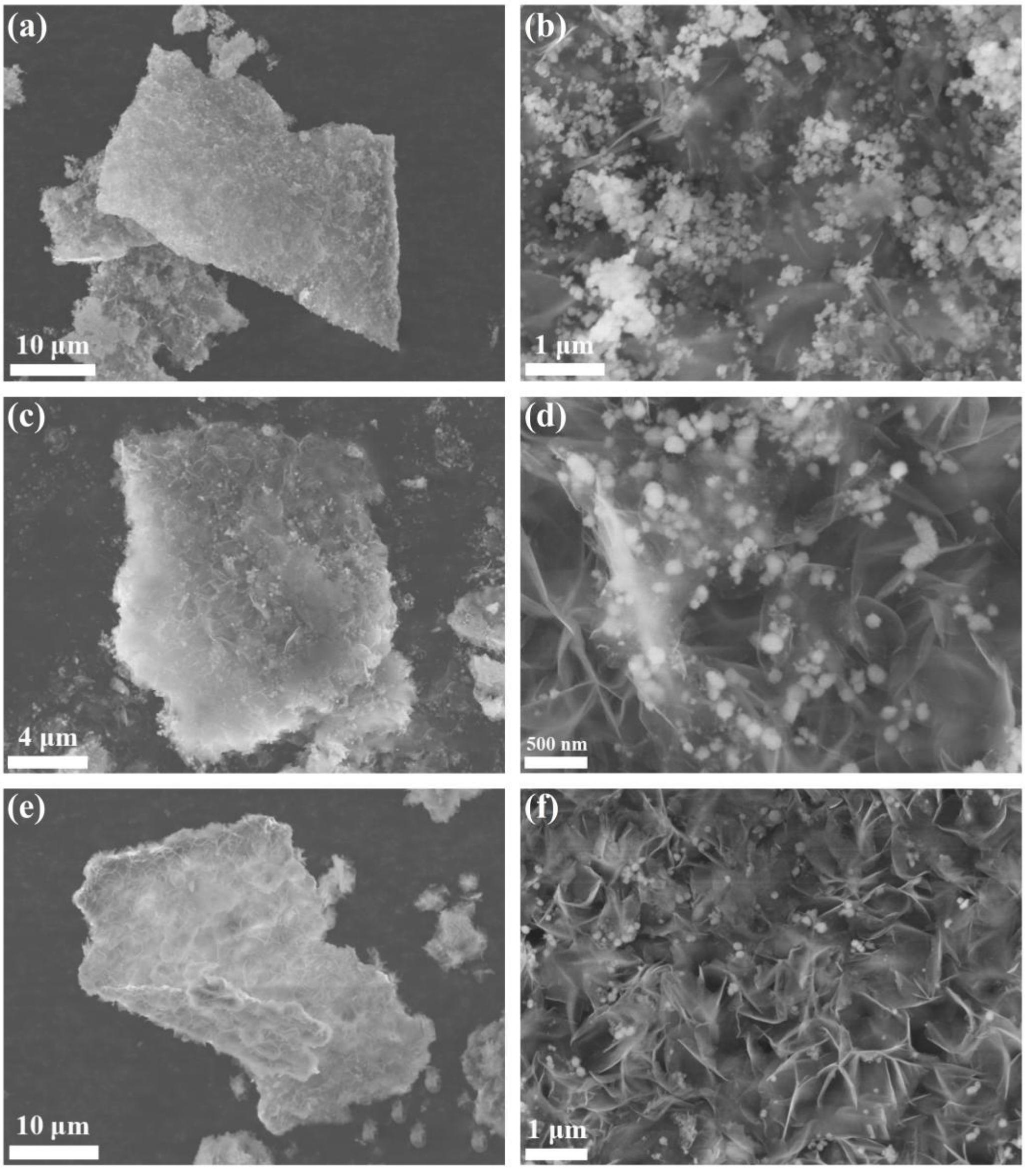

Figure S3 SEM images at different magnifications of (a, b) CoO/MXene-1 composites, (c, d) CoO/MXene-2 composites and (e, f) CoO/MXene-3 composites.

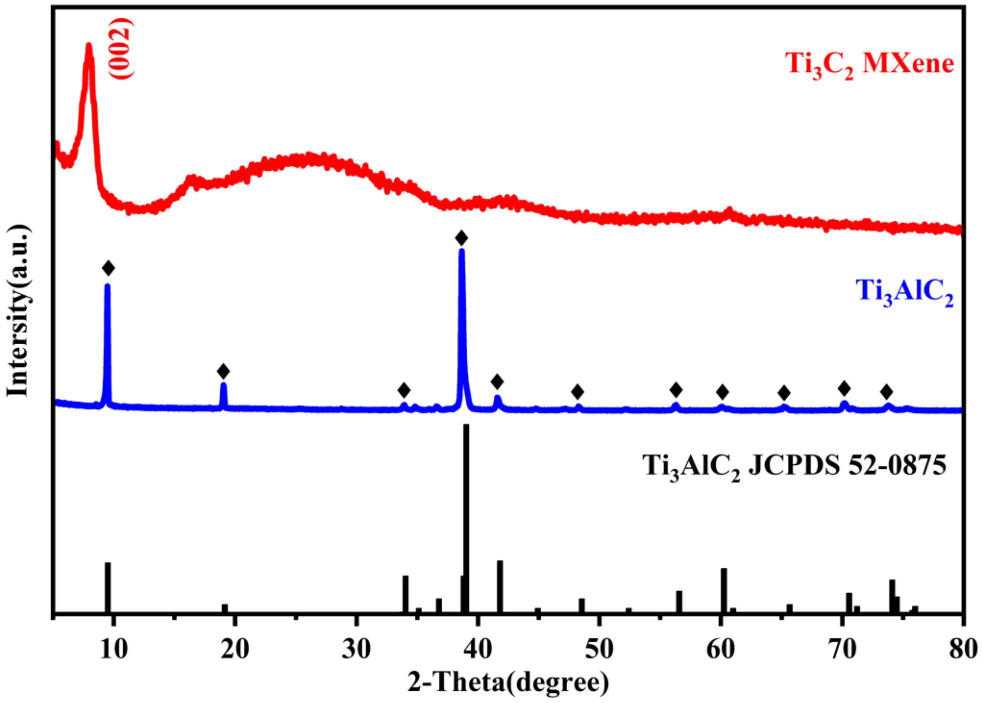

Figure S4 XRD patterns of Ti_3AlC_2 and MXene nanosheets.

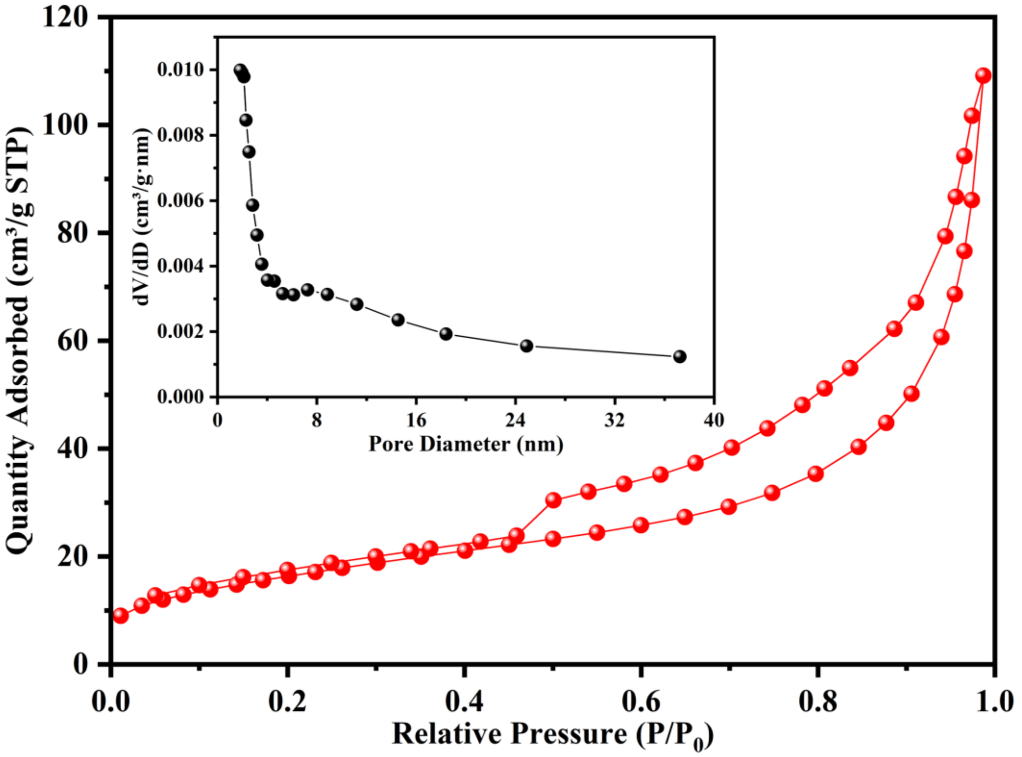

Figure S5 N_2 adsorption/desorption isotherms and the corresponding pore-size distribution profile (inset) of CoO/MXene composites.

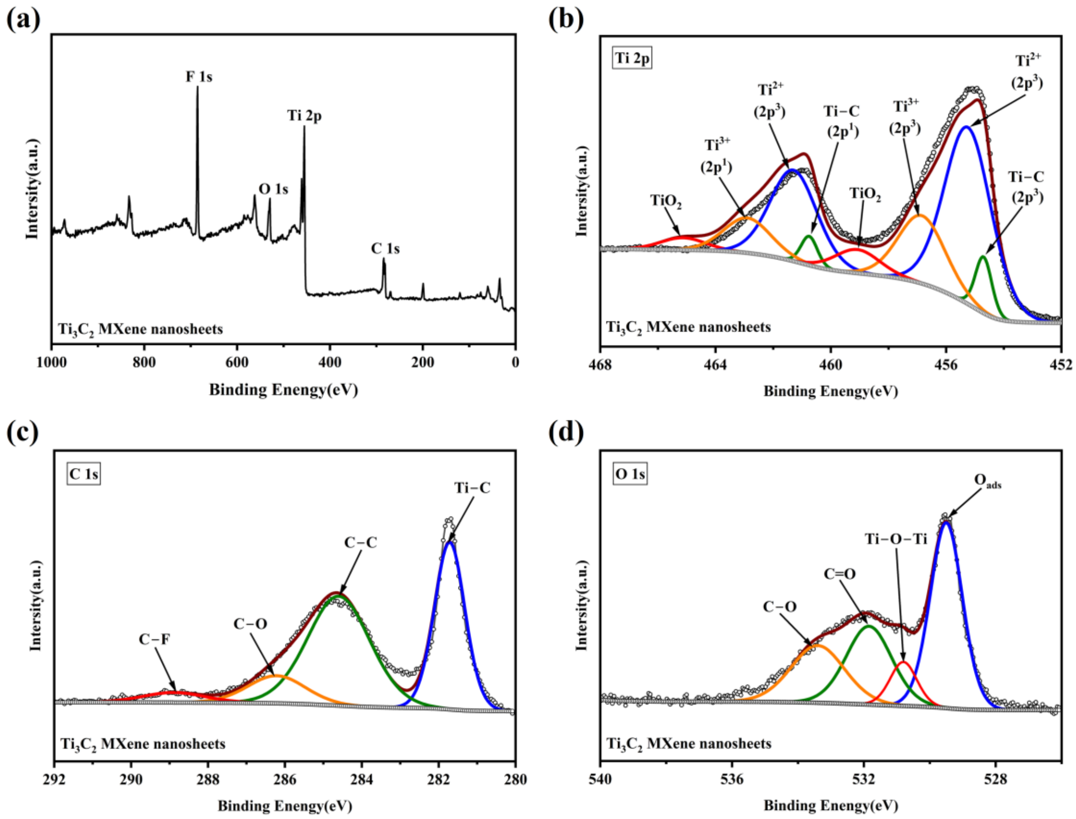

Figure S6 (a) XPS survey spectrum, high-resolution XPS spectra (b) Ti 2p, (c) C 1s, and (d) O 1s of MXene nanosheets.

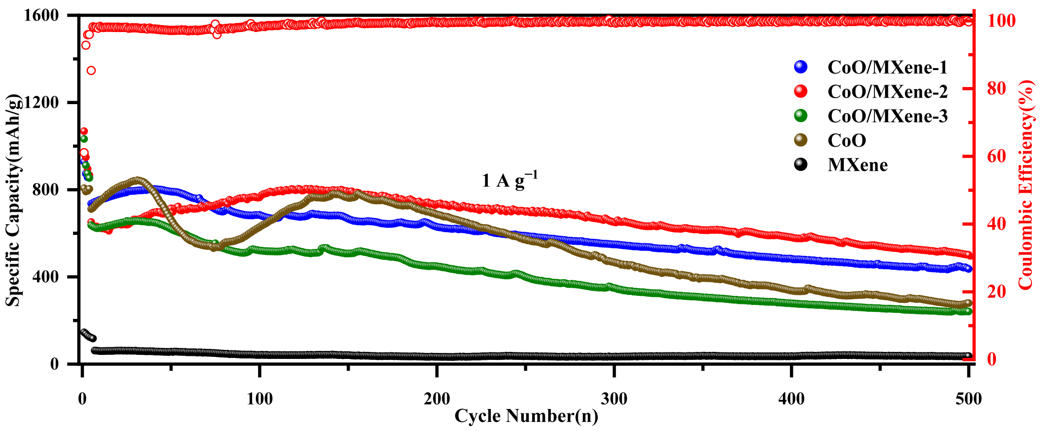

Figure S7 Long cycle performance at 1 A g^{-1} for 500 cycles of CoO/MXene in comparison to CoO, and MXene.

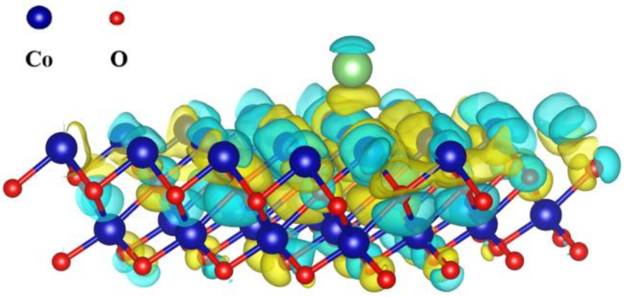

Figure S8 Differential charge density after Li insertion of CoO.

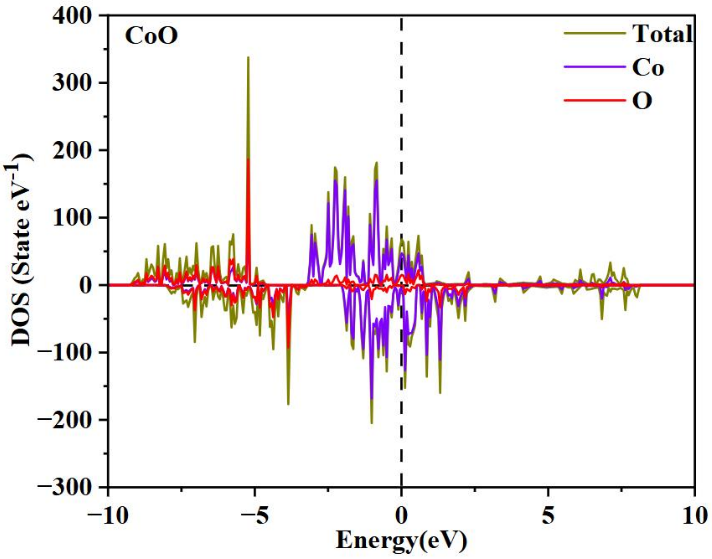

Figure S9 Density of states of CoO material.

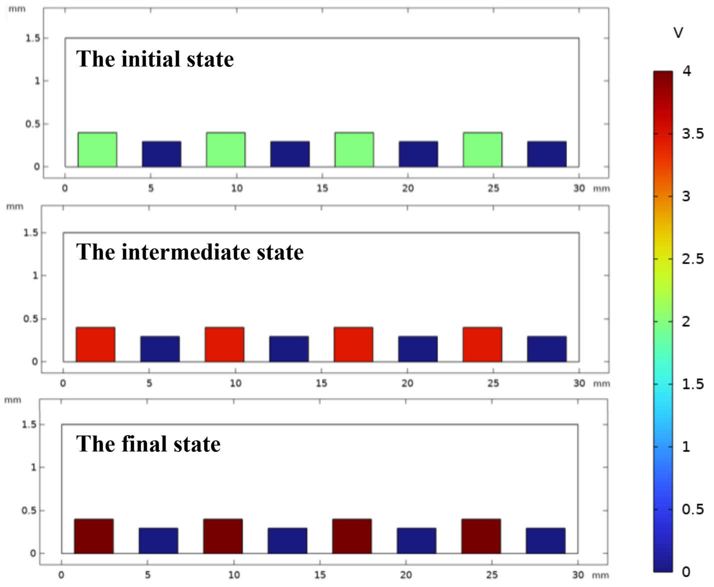

Figure S10 Simulated cell voltage at the initial, intermediate, and final stages of charging.

Table S1. Reversible specific capacities of five electrodes at different current densities

Current Density ($A\ g^{-1}$)	Specific Capacity ($mAh\ g^{-1}$)				
	CoO/MXene-1	CoO/MXene-2	CoO/MXene-3	CoO	MXene
0.1	851.1	1006.6	685.3	926.7	104.1
0.3	782.9	943.6	621.5	854.8	72.3
0.5	766.0	904.6	576.0	815.5	56.2
0.8	725.9	820.7	502.3	745.9	41.1
1.0	704.0	759.2	436.9	704.7	34.4
0.1	1144.8	1115.2	811.8	892.1	77

Table S2 EIS fitting parameters of five electrodes.

Sample	R_s	CPE		R_{ct}	Z_w		
	(ohm)	CPE-T	CPE-P	(ohm)	W-R	W-T	W-P
CoO	4.708	8.659E-06	0.80966	169.3	6.1E-05	1.9761E-08	0.44948
MXene	3.208	1.039E-05	0.85062	43.45	31.75	0.63497	0.34328
CoO/MXene-1	3.394	1.151E-5	0.83221	90.05	3.598	0.0045175	0.47222
CoO/MXene-2	2.919	1.1133E-5	0.8847	60.77	38.81	0.22254	0.38567
CoO/MXene-3	2.894	8.8837E-6	0.82986	106.8	0.17451	0.00016153	0.46757

Table S3 Capacity comparison of present work with reported CoO composites.

sample	Current density (A g^{-1})	Cycle number	Capacity (mAh g^{-1})	References
CoO/Ti_3C_2	0.1	100	324	[1]
CoO/NGA	0.2	200	896	[2]
CNF/CoO	0.2	100	530	[3]
Ti_3C_2@CoO/ZnO	1	300	627	[4]
CNTs@CoO@PC	0.2	300	1090	[5]
CoO-Ns/F	0.2	100	814.7	[6]
CoO@TiO_2@C	0.5	200	1136	[7]
CoO/CF	0.1	100	847	[8]
FeO/CoO	0.3	200	774.9	[9]
Co/CoO/C	0.1	100	557.37	[10]
CoO@SnO_2	0.5	300	509	[11]
CoO/rGO	0.5	500	576.8	[12]
CoO/MXene	0.8	300	731.7	This work

Table S4 Areal capacity comparison of present work with reported anode material

sample	Areal current density (mA cm^{-2})	Areal capacity (mAh cm^{-2})	References
Si/ONT	0.5	0.185	[13]
LFP@GC	0.05	0.398	[14]
TiO2	5	0.347	[15]
Cu2O /WO_3	0.06	0.357	[16]
3DRC-PTSS	0.1	0.107	[17]
CoO/MXene	6	0.45	This work
CoO/MXene	0.6	0.58	This work

References

[1] X. Li, J. Zhu, Y. Fang, W. Lv, F. Wang, Y. Liu, H. Liu, Hydrothermal preparation of CoO/Ti3C2 composite material for lithium-ion batteries with enhanced electrochemical performance, Journal of Electroanalytical Chemistry 817 (2018) 1–8. https://doi.org/10.1016/j.jelechem.2018.03.031.

[2] E. Pan, Y. Jin, Y. Wang, C. Zhao, X. Bo, M. Jia, Facile synthesis of mesoporous 3D CoO/nitrogen-doped graphene aerogel as high-performance anode materials for lithium storage, Microporous and Mesoporous Materials 267 (2018) 93–99. https://doi.org/10.1016/j.micromeso.2018.03.025.

[3] J. Jiang, C. Ma, T. Ma, J. Zhu, J. Liu, G. Yang, Y. Yang, A novel CoO hierarchical morphologies on carbon nanofiber for improved reversibility as binder-free anodes in lithium/sodium ion batteries, Journal of Alloys and Compounds 794 (2019) 385–395. https://doi.org/10.1016/j.jallcom.2019.04.275.

[4] H. Tang, M. Jiang, E. Ren, Y. Zhang, X. Lai, C. Cui, S. Jiang, M. Zhou, Q. Qin, R. Guo, Integrate electrical conductivity and Li+ ion mobility into hierarchical heterostructure Ti3C2@CoO/ZnO composites toward high-performance lithium ion storage, Energy 212 (2020) 118696. https://doi.org/10.1016/j.energy.2020.118696.

[5] Y. Chen, J. Song, Y. Li, Q. Tian, J. Chen, L. Yang, High lithium storage performance of CoO with a distinctive dual-carbon-confined nanoarchitecture, Nanoscale 13 (2021) 12938–12950. https://doi.org/10.1039/D1NR02523F.

[6] L.-H. Wang, X.-L. Teng, Y.-F. Qin, Q. Li, High electrochemical performance and structural stability of CoO nanosheets/CoO film as self-supported anodes for lithium-ion batteries, Ceramics International 47 (2021) 5739–5746. https://doi.org/10.1016/j.ceramint.2020.10.160.

[7] Y.F. Yuan, W.C. Zhao, L. Chen, G.S. Cai, S.Y. Guo, CoO hierarchical mesoporous nanospheres@TiO2@C for high-performance lithium-ion storage, Applied Surface Science 556 (2021) 149810. https://doi.org/10.1016/j.apsusc.2021.149810.

[8] Y. Yuan, H. Li, *In situ* growth of CoO nanosheets on a carbon fiber derived from corn cellulose as an advanced hybrid anode for lithium-ion batteries, New J. Chem. 46 (2022) 18664–18670. https://doi.org/10.1039/D2NJ04039E.

[9] C. Liu, S. Yuan, Y. Yang, X.-X. Zhao, X. Duan, B. Cao, Q. Wang, Prussian blue analogues-derived nitrogen-doped carbon-coated FeO/CoO hollow nanocages as a high-performance anode material for Li storage, Rare Met. 42 (2023) 4070–4080. https://doi.org/10.1007/s12598-023-02373-2.

[10] Y.-J. Song, K.-H. Kim, H.-J. Ahn, Co/CoO particle within F, N-codoped mesoporous carbon framework for anode of lithium-ion batteries, Journal of Alloys and Compounds 969 (2023) 172365. https://doi.org/10.1016/j.jallcom.2023.172365.

[11] W. Liu, Y. An, X. Zhang, L. Wang, C. Li, Y. Xu, X. Zhang, S. Li, S. Yi, Y. Gong, General Synthesis of Graphene/Metal Oxide Heterostructures for Enhanced Lithium Storage Performance. Adv Funct Mater 34 (2024) 2313274. https://doi.org/10.1002/adfm.202313274.

[12] C. Hu, C. Zhou, F. Xia, H. Wang, C. Zhou, Y. Zhang, Q. Tao, Y. Meng, X. Li, Mesoporous CoO@SnO2 yolk–shell nanospheres as enhanced anode materials for lithium-ion batteries. Journal of Energy Storage 111 (2025) 115390. https://doi.org/10.1016/j.est.2025.115390.

[13] R. Jung, J. Yoo, K. Lee, Promising high-durability anode for lithium-ion batteries using optimally sized silicon particles embedded in TiO_2 nanotubes, Journal of Alloys and Compounds 1022 (2025) 180075. https://doi.org/10.1016/j.jallcom.2025.180075.

[14] Y. Zhang, S. Zheng, F. Zhou, X. Shi, C. Dong, P. Das, J. Ma, K. Wang, Z.-S. Wu, Multi-Layer Printable Lithium Ion Micro-Batteries with Remarkable Areal Energy Density and Flexibility for Wearable Smart Electronics, Small 18 (2022) 2104506. https://doi.org/10.1002/smll.202104506.

[15] Y. Jiang, C. Hall, P.A. Burr, N. Song, D. Lau, J. Yuwono, D.-W. Wang, Z. Ouyang, A. Lennon, Fabrication strategies for high-rate TiO2 nanotube anodes for Li ion energy storage, Journal of Power Sources 463 (2020) 228205. https://doi.org/10.1016/j.jpowsour.2020.228205.

[16] Copper foil after hydrothermal treatment in acidified tungstate solution as conductor- and binder-Free anode electrodes for high performance lithium-ion batteries, Instrumentation Science & Technology (n.d.). https://www.tandfonline.com/doi/abs/10.1080/10739149.2021.2021939 (accessed February 3, 2026).

[17] A. Gangadharan, S. Kali, S. Mamidi, A. D. Pathak, C. S. Sharma, Carbon-MEMS based rectangular channel microarrays embedded pencil trace for high rate and high-performance lithium-ion battery application, Materials Advances 2 (2021) 7741 – 7750. https://doi.org/10.1039/D1MA00745A.
